# Use of Recycling-Reflection Color-Purity Enhancement Film to Improve Color Purity of Full-Color Micro-LEDs

**DOI:** 10.1186/s11671-021-03642-8

**Published:** 2022-01-03

**Authors:** Zhi Ting Ye, Jun-Yi Wu

**Affiliations:** Department of Mechanical Engineering, Advanced Institute of Manufacturing with High-Tech Innovations, National Chung Cheng University, 168, University Rd., Min-Hsiung, Chia-Yi, 62102 Taiwan

**Keywords:** Micro-LEDs, Quantum dots, Color purity, Recycling-reflection color-purity-enhancement film, Light conversion efficiency

## Abstract

**Abstract:**

A common full-color method involves combining micro-light-emitting diodes (LEDs) chips with color conversion materials such as quantum dots (QDs) to achieve full color. However, during color conversion between micro-LEDs and QDs, QDs cannot completely absorb incident wavelengths cause the emission wavelengths that including incident wavelengths and converted wavelength through QDs, which compromises color purity. The present paper proposes the use of a recycling-reflection color-purity-enhancement film (RCPEF) to reflect the incident wavelength multiple times and, consequently, prevent wavelength mixing after QDs conversion. This RCPEF only allows the light of a specific wavelength to pass through it, exciting blue light is reflected back to the red and green QDs layer. The prototype experiment indicated that with an excitation light source wavelength of 445.5 nm, the use of green QDs and RCPEFs increased color purity from 77.2% to 97.49% and light conversion efficiency by 1.97 times and the use of red QDs and RCPEFs increased color purity to 94.68% and light conversion efficiency by 1.46 times. Thus, high efficiency and color purity were achieved for micro-LEDs displays.

**Graphical Abstract:**

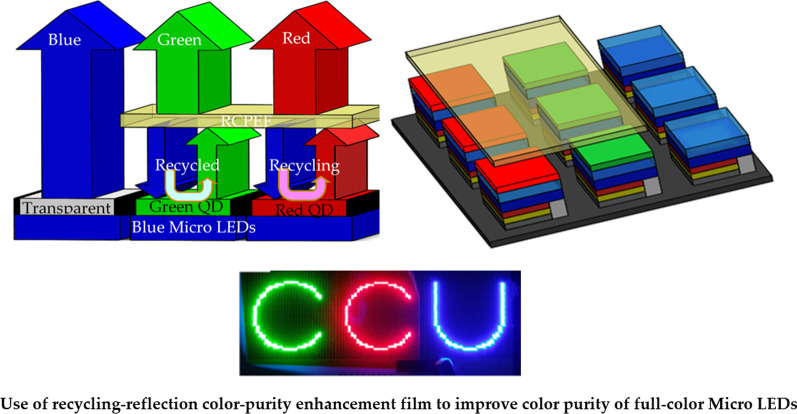

## Introduction

Displays change human reading habits and reduce the use of a lot of paper. All people and every industry need a monitor. Billboards, TV screens, mobile phones, household appliances, and car dashboards all use display applications. Thus, display technical specifications are continually improved through research [1, 2]. The earliest display screens used cathode ray tubes (CRTs), with large size and high power consumption being major drawbacks [3]. Liquid–crystal displays (LCDs), which largely replaced CRTs, are thin and light. However, LCD screens cannot emit light, and thus, the use of backlight and the emission of full-color pixels through color filters are required [4, 5]. CCFL backlight elements contain "mercury (Hg)" toxic substances, the LEDs (light emitting diodes) have just solved the shortcomings of CCFL backlight elements and have become the current mainstream liquid crystal backlight [6, 7]. Light-emitting diodes (LEDs) backlights utilize three types of light sources, with white LEDs (WLEDs) currently used in most displays. To achieve thinness and lightness, edge-lit backlights are often used. When the light guide plate is of poor quality, hot spots tend to appear, causing problems relating to low light uniformity and light extraction efficiency [8, 9]. The second type of light source is a direct-lit backlight, which uses WLEDs and provides more advantages in terms of contrast, brightness, and cost-effectiveness relative to edge-lit backlights [10]. The third light source uses red, green, and blue (RGB) LEDs, but the varying attenuation rates of this light source cause color shift, increase production cost, and impose a high technical threshold [11, 12]. The LCD is still the current mainstream display, but its lack of self-luminosity leads to poor overall control efficiency with respect to the interaction of the backlight with the liquid crystal through the color filter and two polarizers. This drawback has attracted much criticism [13]. In contrast to LCDs, organic LEDs (OLEDs) display technology does not require a backlight and instead uses a thin coating of organic light-emitting materials. The material of the light-emitting layer allows for the three primary colors of red, green, and blue to be produced [14, 15]. Multiple studies have examined OLEDs display technology. Ai et al. proposed an OLEDs display technology based on a combination of perovskite and LEDs, and at an excitation wavelength of 710 nm, its external quantum efficiency (EQE) reached 27% [16]. Chen et al. proposed a high-efficiency red OLEDs display with an EQE of 25.2% at a peak excitation wavelength of 680 nm [17]. Hu proposed a full-color blue organic LEDs with a patterned red–green quantum dots (QDs) color conversion layer and achieved a color gamut standard (BT.2020) of 95% with a 6.6-in full-color display [18]. In contrast to LCDs, OLEDs can emit light and do not require a backlight module. However, due to the characteristics of organic materials, a static image is prone to burn-in after a long period of use. This is the current problem that affects OLEDs [19, 20]. QLEDs (quantum dots light-emitting diodes) utilize QDs display technology. QDs are characterized by wide absorption and narrow emission [21, 22]. Backlight blue LEDs produce excellent red, green, and blue light through QDs films, which provide excellent color saturation performance. However, this technology is classified as a non-self-luminous light source technology [23, 24]. Su et al. explored the use of red, green, and blue transparent QLEDs that are built on a flexible plastic substrate and vertically integrated with UV glue. They reported EQE levels of 12.0%, 8.5%, and 4.5% for red, green, and blue transparent QLEDs, respectively, demonstrating the feasibility of individually controllable RGB QLEDs [25]. Another predecessor of micro-LEDs technology is mini LEDs technology. The advantage of mini LEDs is their small size. Ye et al. proposed a modified package structure for optimizing the light field type of mini LEDs, which can be used as a backlight source for display and lighting [26, 27]. The Scholars have recently conducted extensive research on extremely small RGB micro-LEDs, which all use inorganic semiconductor materials. The material properties of such LEDs grant them the advantages of photoelectric conversion efficiency, high brightness, high reliability, and fast response times [28, 29]. However, their application in large panel screens leads to difficulties in performing the mass transfer and technical problems relating to maintenance. When black spots or color shifts appear on the screen, the replacement of sporadic LEDs is a complicated and time-consuming task [30, 31]. Moreover, compared with blue and green LED chips, red LED chips are prone to generating excessive heat due to their material absorption properties, resulting in poor photoelectric conversion efficiency and low EQE. Consequently, they cannot meet display requirements [32]. Chen et al. applied directional control over RGB micro-LEDs made on a 4-in patterned sapphire substrate to achieve a wide color gamut of 114.4% (National Television System Committee, NTSC) and 85.4% (Rec. 2020) [33]. Qi et al. built a micro-LEDs array on a 0.55-in (400 × 240) gallium nitride substrate with a pixel density of 848 PPI and full width at half maximum (FWHM) of 18.2 nm to produce a high-brightness, high-resolution micro-LEDs display [34]. The aforementioned findings indicate that combining blue micro-LEDs with QDs is currently the best solution for achieving full color. Micro-LEDs have the advantages of high contrast, low power consumption, long life, and fast response time, but they can be improved further [35]. Zhang et al. proposed the use of pure blue double-shell InP/(ZnS) QDs with an emission wavelength of 468 nm and a quantum yield of 45%, and they managed to increase EQE by 2.8 times [36]. Yang Li et al. proposed the application of microfluidic technology combined with a red–green perovskite QDs color conversion layer for use in micro-LEDs displays and achieved a wide color gamut (NTSC) of 131% [37]. Yin et al. combined CsPbBr3 perovskite and CdSe QDs to develop a green–red color conversion layer that achieved a color gamut standard (NTSC) of 129% [38]. Shih et al. suggested the use of QDs composite materials in place of color filters for full-color displays and achieved 86.16% conversion efficiency through the implementation of a QDs color conversion layer [39]. Furthermore, current displays add a color filter to improve color purity, which absorbs most of the wavelength band and only allows specific wavelengths to pass. Thus, light extraction efficiency is substantially compromised [40–42]. Few studies have focused on improving the color purity of micro-LEDs hybrid QDs. Poor color purity results in poor color saturation and reduces the range of the color gamut that can be displayed by a monitor. Therefore, the enhancement of color purity is crucial. The present paper proposes the use of an RCPEF that combines red and green QDs and blue micro-LEDs. This RCPEF only allows the light of a specific wavelength to pass through it, excited blue light is reflected back to the red and green QDs layer, and the corresponding red and green lights are emitted after being absorbed by the QDs, thereby improving color purity and reducing absorption (which leads to substantial light loss). This proposed solution meets display requirements by enabling high conversion efficiency and high color saturation.

## Methods

### Definition of Color Purity

Color purity indicates how close the color of a sample is to its dominant wavelength spectrum and is defined as the ratio of the distance between the chromaticity coordinates of the measured object and CIE1931 center versus the distance between the chromaticity coordinates of the standard light source and CIE1931 center. Color purity can be calculated using Eq. (1). (*x*_*d*_, *y*_*d*_) are the coordinates for the main wavelength spectrum’s color light source, (*x*_*s*_, *y*_*s*_) are the chromaticity coordinates of the measured object, and (*x*_*i*_, *y*_*i*_) are the chromaticity coordinates for the CIE1931 center.

1 $${\text{Color}}\;{\text{purity}} = \frac{{\sqrt {\left( {x_{s} - x_{i} } \right)^{2} + \left( {y_{s} - y_{i} } \right)^{2} } }}{{\sqrt {\left( {x_{d} - x_{i} } \right)^{2} + \left( {y_{d} - y_{i} } \right)^{2} } }} \times 100$$

### Definition of Light Conversion Efficiency

Conversion efficiency refers to the ratio of effective output energy to input energy. Light conversion efficiency can be obtained using Eq. (2):

2 $${\text{Light}}\;{\text{conversion}}\;{\text{efficiency}}: = \frac{{{\text{Area}}_{{\left( {{\text{g}},\;{\text{r}}} \right) \;{\text{or}}\; \left( {{\text{G}},\;{\text{R}}} \right)}} }}{{{\text{Area}}_{{\text{B}}} + {\text{Area}}_{{\left( {{\text{g}},\;{\text{r}}} \right) \;{\text{or}}\; \left( {{\text{G}},\;{\text{R}}} \right)}} }} \times 100\%$$

where Area_(g, r)_ is the total radiated power in the red and green bands without RCPEF, Area_(G, R)_ is the total radiated power in the red and green bands with a layer of RCPEF, and Area_B_ is the total radiated power in the blue band (all units are in mW).

### Preparation Process for Red and Green Quantum Dots

**Fig. 1:**
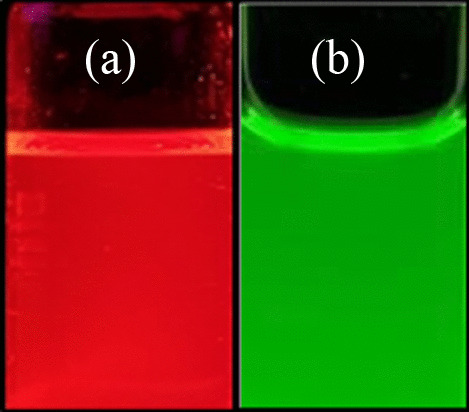
Samples of **a** red and **b** green QDs

The quantum dots used in this study are red and green CdSe/ZnS, the main reaction materials are cadmium oxide (CdO), zinc oxide (ZnO), silicon (Se), trioctyl phosphine oxide (TOP), lauric acid (LA, C_12_H_24_O_2_), and n-hexane (C_6_H_14_). Hexadecylamine (HDA) is used to prevent the agglomeration reaction of QDs, methanol (CH_3_OH) is used to prevent the agglomeration reaction, and argon (Ar) is used throughout the process to ensure environmental vacuum. The first part of the production process involves mixing 0.0049 g CdO, 0.0308 g ZnO, and 1.7495 g LA in a three-necked flask, then pour argon and stir the mixture with a magnet and heat it to 230 °C. After reaching 230 °C, the mixture was naturally cooled to 30 °C. The cooling time is used to prepare the precursor. The preparation involves injecting 0.0948 g of Se into TOP and n-hexane to form a mixture, and then shaking to completely dissolve the Se in TOP and n-hexane. Cool the main reactant to 30 °C, add 3.2505 g HDA, heat the mixture to 230 °C, and heat the red QDs to 300 °C, and the green QDs to 320 °C. Then the prepared precursor is added and the reaction time is controlled. The reaction time between the red light quantum dots and the precursor is 60 s, and the reaction time between the green light quantum dots and the precursor is 3 s. After the reaction was completed, the mixture was quickly cooled to 150 °C, and methanol heated to about 60 °C was added to terminate the agglomeration reaction. Finally, centrifuge at 5000 rpm for 10 min, pour out the methanol, and air dry. Samples of red and green QDs are shown in Fig. 1. Figure 1a shows a red light CdSe/ZnS QDs sample, and Fig. 1b shows a green light CdSe/ZnS QDs sample. Both red and green QDs are mixed in n-hexane solution.

**Fig. 2:**
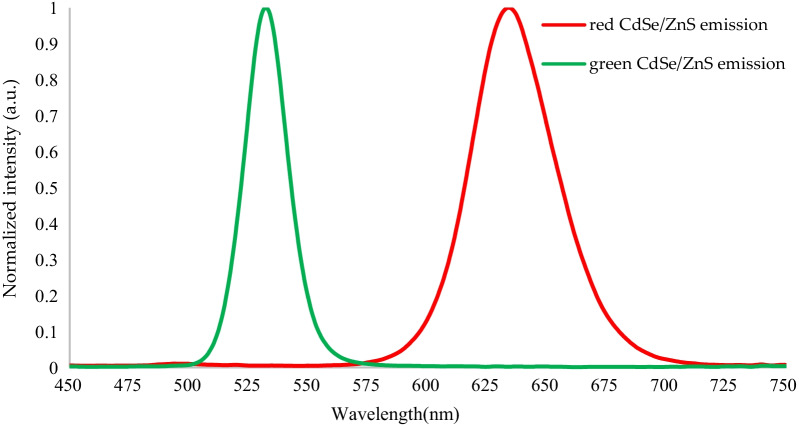
Schematic diagram of the normalized spectral radiance of red and green CdSe/ZnS

The normalization spectral of the red and green CdSe/ZnS quantum dots are shown in Fig. 2. The emission wavelength and FWHM of the red CdSe/ZnS quantum dots are 633.5 and 41.5 nm. The emission wavelength and FWHM of the green CdSe/ZnS quantum dots are 531.5 and 22 nm.

The specifications of the red and green CdSe/ZnS QDs samples are shown in Table 1. The measurement results of the photoluminescence (PL) spectrometer show that the emission wavelength and FWHM of the red CdSe/ZnS QDs are 633.5 and 41.5 nm, and the emission wavelength and FWHM of the green CdSe/ZnS QDs are 531.5 and 22 nm. The concentration of the red and green CdSe/ZnS QDs samples is 20 wt%. By controlling the reaction time of the main reactant CdO and the precursor Se, the particle size of the red CdSe/ZnS QDs is 4.4 nm and the particle size of the green CdSe/ZnS QDs is 3.3 nm.

**Table 1:** Specifications of red and green CdSe/ZnS samples

	Red CdSe/ZnS	Green CdSe/ZnS
Wavelength (nm)	633.5 nm	531.5 nm
FWHM (nm)	41.5 nm	22 nm
Concentration weight (wt%)	20 wt%	20 wt%
Particle diameters (nm)	4.4 nm	3.3 nm

### RCPEF Principle and Optimal Design

**Fig. 3:**
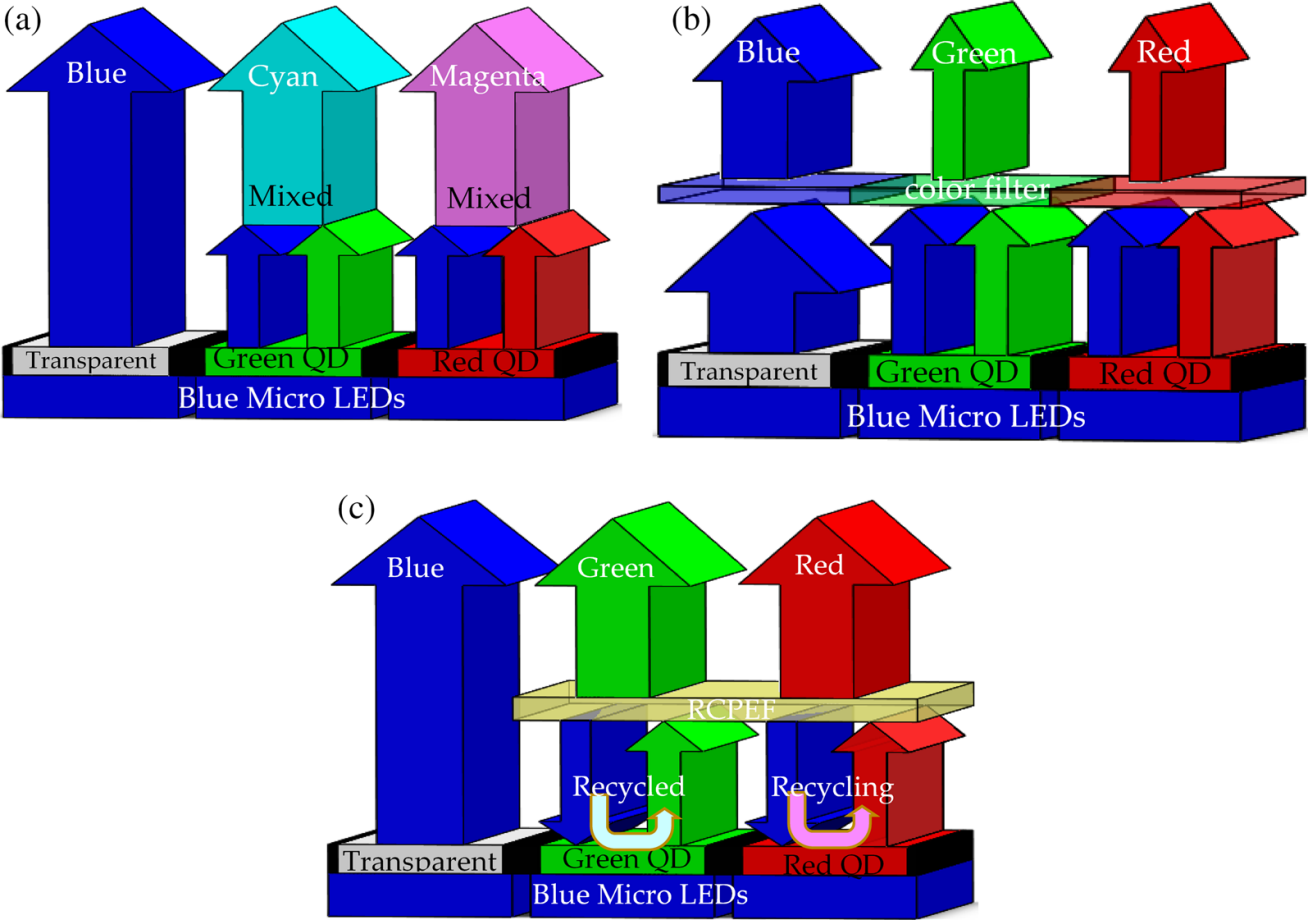
RCPEF color purity improvement principle: **a** no RCPEF light emission, **b** traditional color filter architecture, and **c** combined RCPEF light emission architecture

We propose the use of RCPEF to achieve high color purity. When the excited blue light wavelength emitted by an LEDs passes through red and green QDs, the inability of the QDs to completely absorb the incident wavelength means that the total emission wavelength is the emission wavelength of the incident wavelength of the mixed QDs (Fig. 3a), which leads to poor color purity and color cast problems. The traditional solution is to add a color filter (CF) layer and apply the principle of material absorption to absorb the blue light, such that the corresponding green and red pixels only emit green and red light, respectively. Although a higher color purity can be obtained, a considerable amount of energy is lost due to material absorption as presented in Fig. 3b. To address this problem in the present study, a layer of RCPEF was added above the red-green QDs of the color conversion layer. As shown in Fig. 3c, when part of the incident wavelength (blue light) passed through the RCPEF, the RCPEF reflected the blue band back to the QDs color conversion layer, and the red and green QDs absorbed again to re-excite and re-emit red and green wavelengths. After several cycles, relatively pure RGB colors were obtained.

**Fig. 4:**
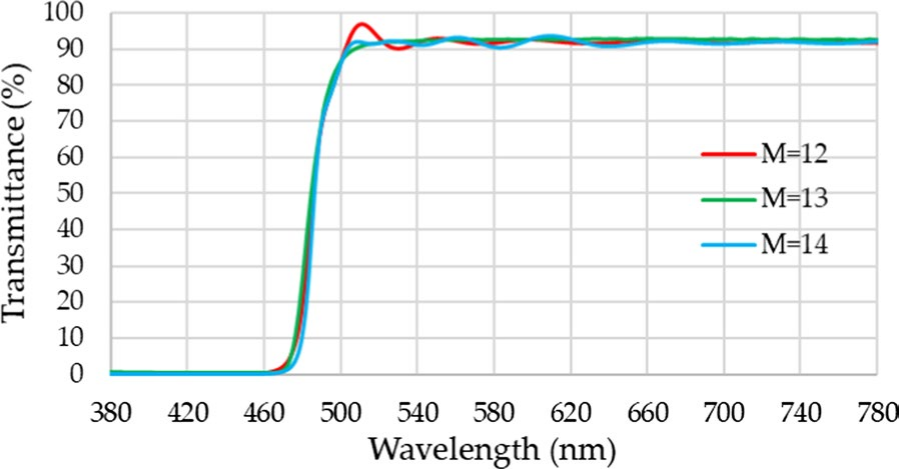
RCPEF bandpass film simulation

In the present study, Essential Macleod (Thin Film Center Inc.) simulation software was used to design the recovery of reflected color purity to enhance the film. The optimal design was achieved using the formula Air/(HL)^M^/SUB of the membrane stack, where L is the low refractive index material SiO_2_, H is the high refractive index material TiO_2_, M is the constant (i.e., the power of the membrane stack), and SUB is the substrate material (i.e., glass). The optimization calculations indicated that when *M* = 12, the 500 nm waveband size ripple was large and the difference between an M of 13 and 14 was small. Therefore, better results can be obtained when *M* = 13. The overall physical thickness was 1442.02 nm. After optimization (Fig. 4), the wavelength band was less than 470 nm for the high reflection area with a penetration rate less than 1%, and the wavelength band was greater than 510 nm for the high penetration area with a penetration rate greater than 92%.

### Structure of High-Color-Purity Full-Color Micro-LEDs

**Fig. 5:**
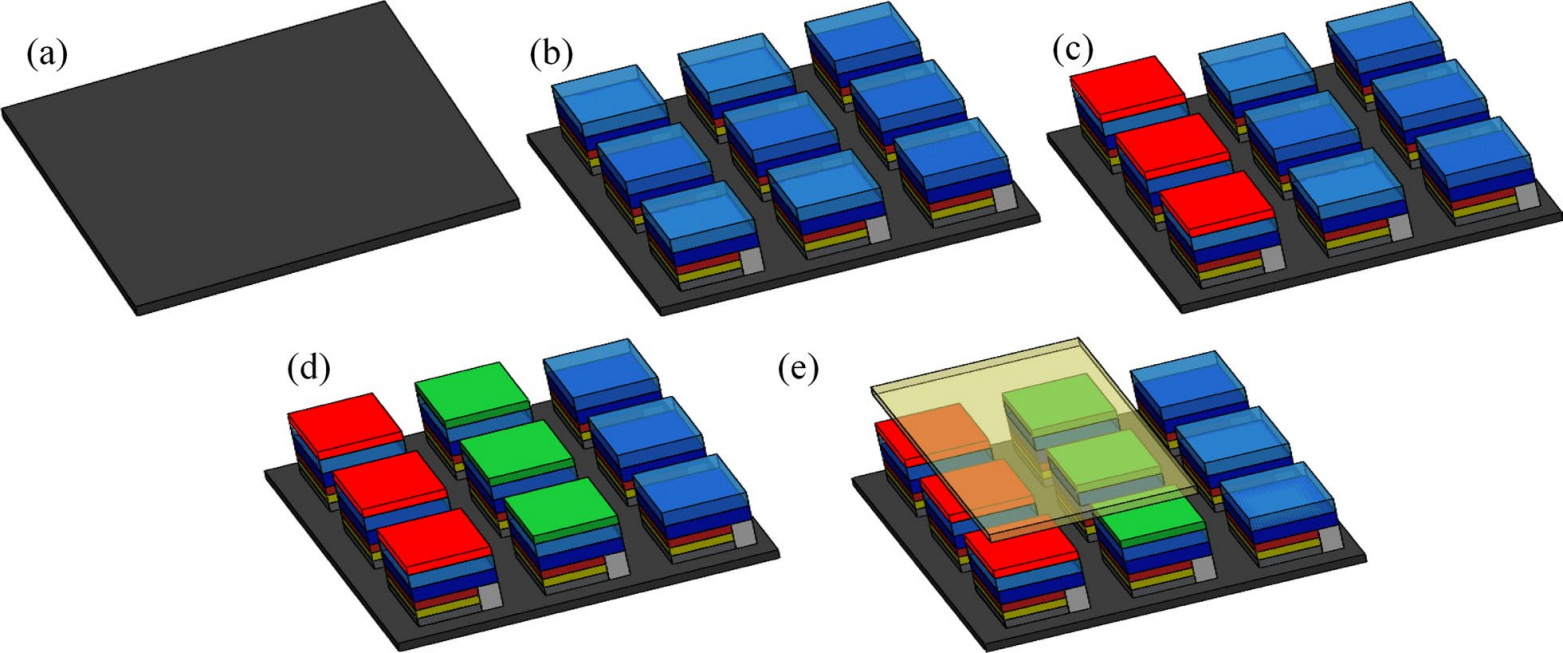
Structure of high-color-purity full-color micro-LEDs: **a** flexible FR4 substrate, **b** die-bonding of blue micro-LEDs on FR-4, **c** dispensation of red-light QDs layer, **d** dispensation of green-light QDs layer, **e** and completion of high-color-purity full-color micro-LEDs with RCPEF

The processing steps and structure of high-color-purity full-color micro-LEDs are shown in Fig. 5. Figure 5a shows a flexible FR4 flexible substrate. (FR-4 is a composite material made from a woven fiberglass cloth and epoxy resin binder.) First, blue micro-LEDs were die-bonded onto the FR4 substrate (Fig. 5b). A layer of CdSe red and green QDs was then applied on specific pixels through dispensation (Fig. 5c, d), and a layer of RCPEF was then added above the red and green QDs pixels (Fig. 5e).

## Results and Discussion

### Blue Micro-LEDs Chip

**Fig. 6:**
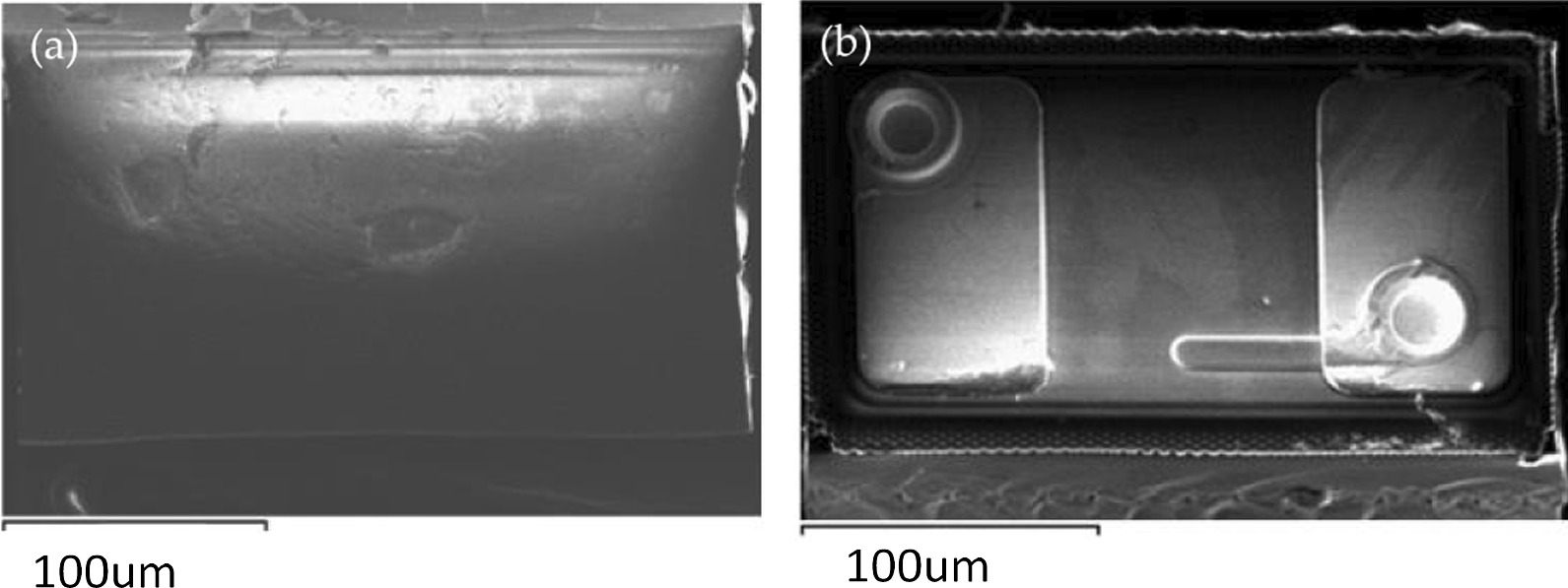
Images of micro-LEDs obtained using a scanning electron microscope. **a** top and **b** bottom views

The blue LEDs chip had a flip-chip structure, and its length, width, and height were 140, 240, and 100 μm, respectively. Figure 6 shows the images of the micro-LEDs that were obtained using a scanning electron microscope.

**Fig. 7:**
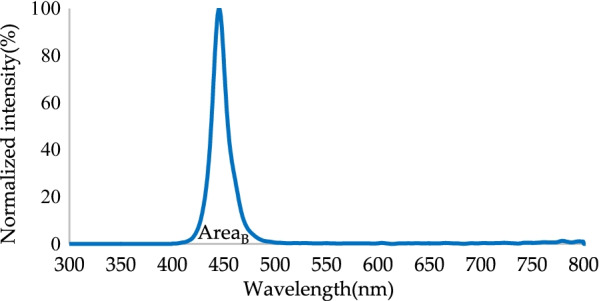
Normalized spectrum of blue micro-LEDs chip

The normalized spectrum of the light source of the blue micro-LEDs chip is shown in Fig. 7. The wavelength peak and FWHM were 445.5 and 16 nm, and the color purity was 98.23%.

### Measurement Data of RCPEF

**Fig. 8:**
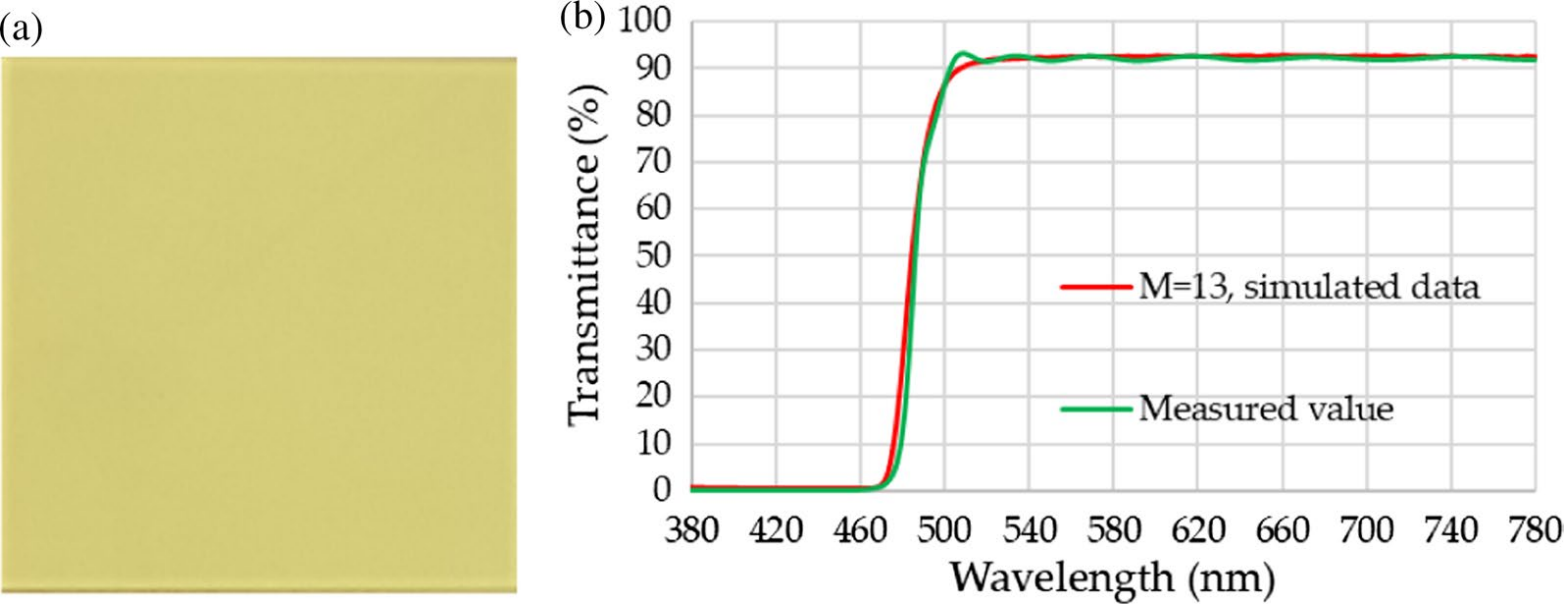
RCPEF sample: **a** prototype sample, **b** measured value of penetration rate of RCPEF

Figure 8 shows the RCPEF sample and presents its measurement data. Figure 8a shows the sample, which measures 5 cm × 5 cm. Figure 8b presents the comparison of the simulated and measured data of the RCPEF, indicating a high consistency between the simulated and measured transmittance rates.

### RCPEF for Green QDs

**Fig. 9:**
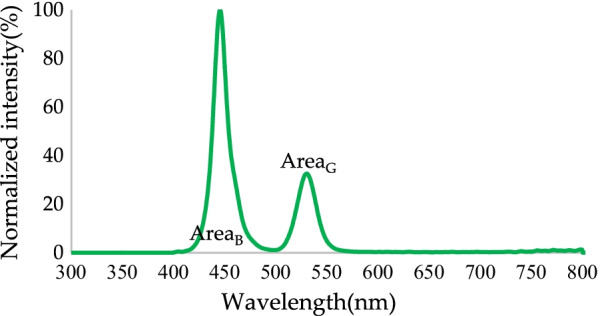
Normalized blue light without RCPEF conversion to green spectrum by QDs

The blue micro-LEDs were converted from green QDs to green light. The normalized spectrum is shown in Fig. 9. The peak emission of green light was 531.5 nm, and the FWHM was 22 nm. Most of the light emitted at this point was radiant blue light. The green light conversion efficiency was only 27.08%, and the color purity was 77.2%. Notably, the color purity at this point was greatly affected by blue light, such that the color conversion efficiency, color purity, and light conversion efficiency were not ideal and a clear color shift was observed.

**Fig. 10:**
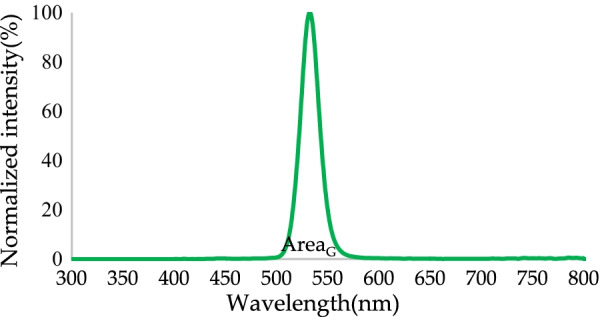
Conversion of normalized blue light to a green spectrum by QDs after addition of RCPEF

After the addition of the RCPEF, a normalized blue-to-green spectrum was achieved (Fig. 10). The light green emission peak was 532 nm, and the FWHM was 21 nm. Some incident wavelengths that could not be absorbed by the QDs were radiated again after being reflected to the QDs layer by the RCPEF. The light conversion efficiency of green light was 53.43%, which was 1.97 times higher than the light conversion efficiency that was observed when the RCPEF was not used. At this point, the color purity could be increased to 97.49%.

**Fig. 11:**
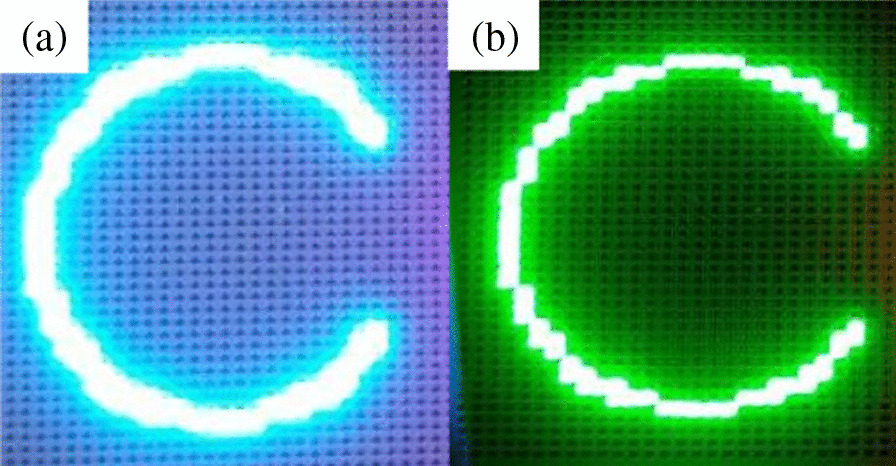
Green QDs sample **a** before and **b** after addition of RCPEF

The data for the blue-to-green light conversion are shown in Fig. 11. Figure 11a shows the color of the emitted light without RCPEF. Because most of the emitted light was in the incident blue wavelength band, the color of the emitted light was the color mixed by the green quantum mixing incident wavelength, which led to a color shift that was similar to cyan. The addition of the RCPEF changed the color of the emitted light (Fig. 11b). The part of the incident wavelength that the QDs could not absorb was reflected by the RCPEF back to the QDs layer for reabsorption and re-excitation, and it was then radiated as green light to obtain purer green light. At this point, the color purity could be increased to 97.49%.

### RCPEF for Red QDs

**Fig. 12:**
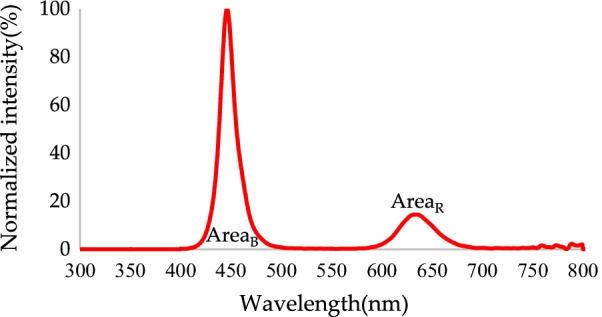
Normalized blue light without RCPEF conversion to red spectrum by QDs

Blue micro-LEDs were converted into red light through red QDs. The data for the conversion of normalized blue light to the red light spectrum are shown in Fig. 12. The peak of red light was 633.5 nm, and the FWHM was 41.5 nm. Most of the emitted light was incident light blue, and at this point, the light conversion efficiency was only 21.78%. Moreover, the color purity value was not indicative because most of the wavelength band that contributed to this value was the light emitted by the incident light-blue light and not that of the red light. Therefore, the resulting color purity and light conversion efficiency were not ideal, and a clear color shift phenomenon was observed.

**Fig. 13:**
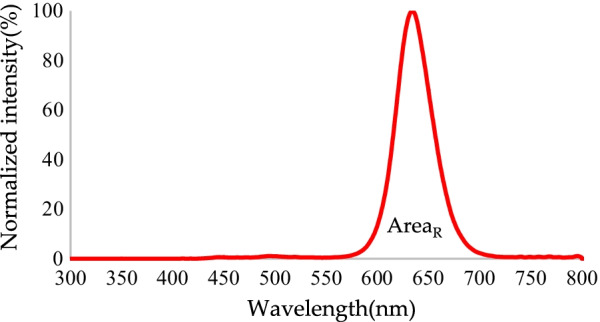
Conversion of normalized blue light to red light by QDs after addition of RCPEF

After the addition of the RCPEF, a normalized blue-to-red spectrum was achieved (Fig. 13). The peak emission of red light was 634 nm, and the FWHM was 40.5 nm. Some incident wavelengths that could not be absorbed by the QDs were reflected by the RCPEF to the QDs layer for reabsorption, re-excitation, and re-emission as red light. The light conversion efficiency of red light was increased to 31.77%, which was 1.46 times higher than light conversion efficiency that was observed before the addition of the RCPEF, and the color purity was increased to 94.68%.

**Fig. 14:**
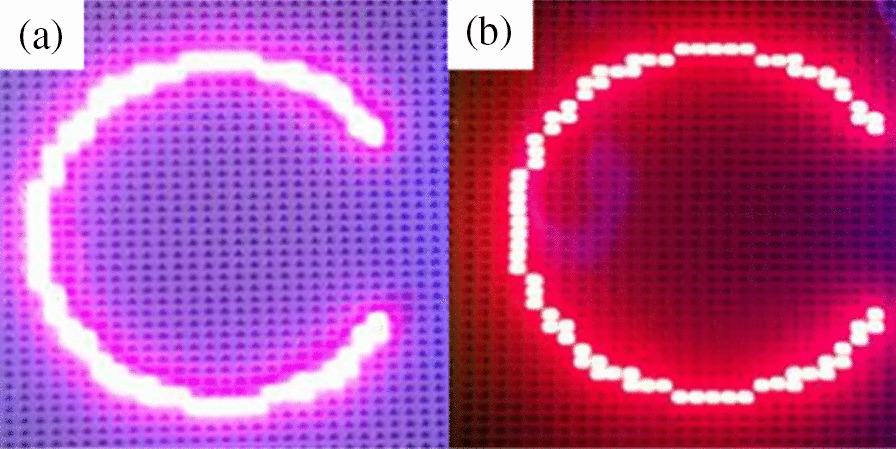
Red QDs sample **a** before and **b** after addition of RCPEF

Figure 14 presents a practical example of the conversion of blue light to red light. Figure 14a shows the color of the emitted light before the addition of the RCPEF. Because most of the emitted light was in the incident blue light band, the light conversion efficiency for red light was only 21.78%. Therefore, the color of the obtained emitted light was red converted by mixing the QDs with the incident wavelength, and its color shift was similar to a magenta color. After the addition of the RCPEF, the color of the emitted light shown in Fig. 14b was achieved. Some incident wavelengths that could not be absorbed by the QDs were reflected by the RCPEF to the QDs layer to be reabsorbed and radiated as red light after excitation. A purer red light could be obtained. The color purity was increased to 94.68%.

**Fig. 15:**
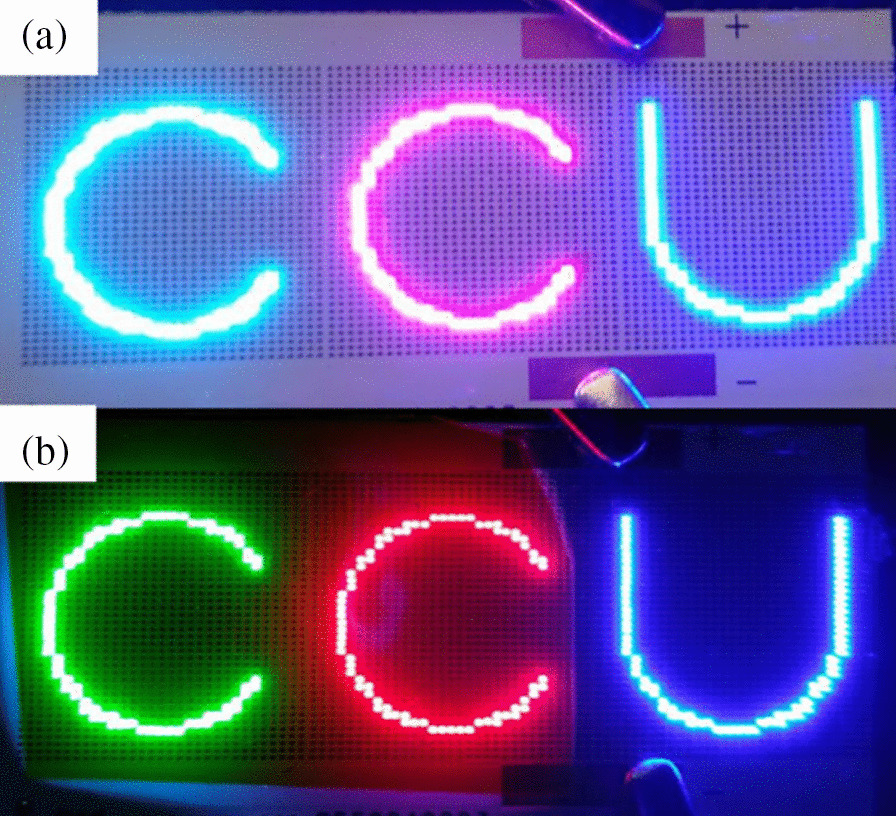
Micro-LEDs colors **a** before and **b** after addition of RCPEF

Figure 15 shows the color purity of the micro-LEDs sample. The light emitted by the sample after the addition of the RCPED is shown in Fig. 15b—compared with the image in Fig. 15a, the light color of the sample exhibits purer RGB colors. Red, green, and blue color purity were 94.68%, 97.49%, and 98.23%.

Table 2 presents the comparison of the color purity and light conversion efficiency of blue micro-LEDs with differing configurations (i.e., with or without RCPEF and with or without red or green QDs). After the addition of the RCPEF, the color purity of red and green QDs was increased to 94.68% and 97.49%. The light conversion efficiency of red and green QDs was increased by 1.46 and 1.97 times. The experimental results indicated that the combination of the blue micro-LEDs with red and green QDs and the RCPEF effectively improved color purity and light conversion efficiency.

**Table 2:** Comparison of blue micro-LEDs with differing configurations

Type	Only blue micro-LEDs	Blue micro-LEDs with G-QDs		Blue micro-LEDs with R-QDs	
RCPEF	Without filter	Without RCPEF	With RCPEF	Without RCPEF	With RCPEF
Light conversion efficiency (%)	100%	27.08%	53.43%	21.78%	31.77%
Peak wavelength (nm)	445.5 nm	531.5 nm	532 nm	633.5 nm	634 nm
FWHM (nm)	16 nm	22 nm	21 nm	41.5 nm	40.5 nm

## Conclusions

This research suggests that combining blue micro-LEDs with red and green QDs and a layer of RCPEF can improve color purity and light conversion efficiency. The incident blue-light wavelengths that cannot be completely absorbed by the QDs are reflected to the color conversion layer. Subsequently, they are reabsorbed by red and green QDs for re-excitation and radiation, thereby achieving high color purity and high conversion efficiency. Thus, the problem of poor color purity in color conversions between micro-LEDs and QDs is alleviated.

Furthermore, because RCPEF is a type of feedback reflection, conversion efficiency can be improved. The prototype experimental data indicate that with the addition of an RCPEF layer, the color purity of the red and green quantum dots increased to 94.68% and 97.49%, respectively, and the light conversion efficiency of the red and green quantum dots increased by 1.46 times and 1.97 times, respectively. The addition of the RCPEF effectively improves the color purity and light conversion efficiency of light such that micro-LEDs displays can achieve higher color saturation and light output efficiency, thus enhancing their display specifications and commercial value. In the future, the application of micro-patterning in ultra-high-pixel micro-LEDs displays can be further examined.

## Data Availability

The datasets supporting the conclusions of this article are available in the article.

## Abbreviations

B: Blue
CF: Color filter
CRT: Cathode ray tube
EQE: External quantum efficiency
FWHM: Full width at half maximum
G: Green
HDA: HEXADECYL amine
LA: Lauric acid
LCD: Liquid–crystal display
LEDs: Light-emitting diodes
NTSC: National television system committee
OLEDs: Organic light-emitting diode
QDs: Quantum dots
QDF: Quantum dots film
QLEDs: Quantum dots light-emitting diodes
R: Red
RCPEF: Reflection color purity enhancement film
SEM: Scanning electron microscope
TOP: Trioctylphosphine
W: White
